# Comparison of Sigma metrics computed by three bias estimation approaches for 33 chemistry and 26 immunoassay analytes

**DOI:** 10.1515/almed-2022-0095

**Published:** 2023-07-04

**Authors:** Şerif Ercan

**Affiliations:** Department of Medical Biochemistry, Lüleburgaz State Hospital, Lüleburgaz Devlet Hastanesi İstiklal Mah, Kırklareli, Türkiye

**Keywords:** bias, chemistry, external quality assurance, immunoassays, internal quality control, Sigma metric

## Abstract

**Objectives:**

Sigma metric can be calculated using a simple equation. However, there are multiple sources for the elements in the equation that may produce different Sigma values. This study aimed to investigate the importance of different bias estimation approaches for Sigma metric calculation.

**Methods:**

Sigma metrics were computed for 33 chemistry and 26 immunoassay analytes on the Roche Cobas 6000 analyzer. Bias was estimated by three approaches: (1) averaging the monthly bias values obtained from the external quality assurance (EQA) studies; (2) calculating the bias values from the regression equation derived from the EQA data; and (3) averaging the monthly bias values from the internal quality control (IQC) events. Sigma metrics were separately calculated for the two levels of the IQC samples using three bias estimation approaches. The resulting Sigma values were classified into five categories considering Westgard Sigma Rules as ≥6, <6 and ≥5, <5 and ≥4, <4 and ≥3, and <3.

**Results:**

When classifying Sigma metrics estimated by three bias estimation approaches for each assay, 16 chemistry assays at the IQC level 1 and 2 were observed to fall into different Sigma categories under at least one bias estimation approach. Similarly, for 12 immunoassays at the IQC level 1 and 2, Sigma category was different depending on bias estimation approach.

**Conclusions:**

Sigma metrics may differ depending on bias estimation approaches. This should be considered when using Six Sigma for assessing analytical performance or scheduling the IQC events.

## Introduction

A vast majority of medical diagnosis and treatments, estimated 60–70 %, are based on data from clinical laboratories [1]. Therefore, the activities of total testing process, which are carried out in three phase namely pre-analytical, analytical, and post-analytical, should be closely observed in order to ensure patient safety. Internal and external quality assurance procedures are routinely performed to monitor the accuracy and precision of the analytical testing process. The analytical performance of a new or existing system can be also evaluated by additional approaches e.g., six Sigma [2].

Six Sigma methodology was introduced into laboratory medicine in 2001, and its implementations extended beyond the acceptability of method [3]. With Sigma metrics, laboratorians can define the internal quality control (IQC) rules, and the number of control measurements required per run, and now apply a risk-based IQC plan by determining control frequency [2].

Six Sigma methodology combines three elements, namely, imprecision, bias, and total allowable error (TEa) into a single value [2]. Multiple sources for the TEa targets exist worldwide. In consensus statement from the 1st Strategic Conference of the European Federation of Clinical Chemistry and Laboratory Medicine (EFLM), three models have been recommended to set analytical performance specifications (APS): the first model based on clinical outcomes, the second on biological variation, and the third on state-of- the-art [4]. The TEa values from different sources for any measurand may be different, which produces a challenge for laboratories in choosing the quality specifications for the Sigma metric calculation [5].

Similar to TEa targets, there is heterogeneity in estimating imprecision for Sigma metric calculation. To obtain the imprecision data, some studies have performed the replication study following a standardized protocol in a relatively brief period [6], [7], [8], [9], [10]. In other studies, the imprecision has been computed using data coming from long-term IQC studies performed routinely [11], [12], [13], [14], [15].

Another challenge for Sigma metric calculation is bias estimation. Bias can be determined from different approaches. In an ideal manner, the preferred approach for bias estimation is to compare the results from a reference material or patient specimens being assayed using a field method with those of a reference method [16]. In previous studies regarding Six Sigma in the clinical laboratory, the bias of a given test has usually been obtained through comparison with the group mean of the external quality assurance (EQA) program. In this approach, multiple bias values obtained from long-term EQA studies were converted into one bias value through averaging [11], [12], [13, 15] or regression analysis [6, 17] for Sigma calculation. Another preferred approach to determine bias is to calculate the difference between the observed control results and the IQC target (or mean) value [5, 10, 14, 15]. Furthermore, some studies have estimated bias through the regression analysis of the results from method comparison studies [7], [8], [9].

The previous studies reported that the Sigma values directly depend on TEa goals from different sources [5, 15, 18]. However, there is limited data on whether Sigma values vary to the preferred approach for estimating bias.

This study aimed to compare Sigma values of 33 chemistry and 26 immunoassay analytes determined using three bias estimation approaches, including averaging the monthly bias values obtained from the EQA studies during a 1-year period, calculating the bias values from regression equation derived from the EQA data, and averaging the monthly bias values from the IQC studies according to the target control values.

## Materials and methods

This observational study was conducted based on the EQA and IQC performance of 59 analytes, 33 for chemistry and 26 for immunoassays, on the Cobas 6000 analyzer (Roche Diagnostics, Manheim, Germany). The analytes evaluated are listed in Table 1.

**Table 1:** The total allowable error (TEa) limits and its sources.

Analyte	The source of the TEa goal	TEa, %
Albumin	CLIA	8.0
Alkaline phosphatase (ALP)	SEQCML BV database	12.0
Alanine aminotransferase (ALT)	EFLM BV database	16.1
Amylase	EFLM BV database	13.2
Anti-Streptolysin O (ASO)	^a^	10.0
Aspartate aminotransferase (AST)	EFLM BV database	13.6
Bilirubin, direct	RCPA	20.0
Bilirubin, total	RCPA	12.0
C reactive protein (CRP)	RiliBAK	20.0
Calcium	RiliBAK	10.0
Chloride	RiliBAK	8.0
Cholesterol, high density lipoprotein	NCEP	13.0
Cholesterol, low density lipoprotein	NCEP	12.0
Cholesterol, total	NCEP	8.9
Creatine kinase	EFLM BV database	22.6
Creatinine	EFLM BV database	7.4
D-dimer	SEQCML BV database	28.0
Gamma-glutamyl transferase (GGT)	EFLM BV database	18.9
Glucose	EFLM BV database	6.5
Hemoglobin A1c	IFCC task force	6.9
Iron	CLIA	15.0
Lactate dehydrogenase (LDH)	EFLM BV database	7.7
Lipase	EFLM BV database	14.2
Lithium	CLIA	15.0
Magnesium	RCPA	8.0
Phosphorus	EFLM BV database	9.7
Potassium	RiliBAK	8.0
Protein, total	CLIA	8.0
Rheumatoid factor	SEQCML BV database	13.5
Sodium	RiliBAK	5.0
Triglyceride	NCEP	15.0
Urea	EFLM BV database	17.8
Uric acid	EFLM BV database	12.8
25-OH Vitamin D	EFLM BV database	12.4
Alpha feto protein	EFLM BV database	17.6
Cancer antigen 125 (CA 125)	EFLM BV database	13.9
Cancer antigen 15-3 (CA 15-3)	RiliBAK	24
Carbohydrate antigen 19-9 (CA 19-9)	EFLM BV database	17.9
Carcinoembryonic antigen (CEA)	EFLM BV database	20.5
Ferritin	SEQCML BV database	16.9
Folate	EFLM BV database	19.7
Free T3	EFLM BV database	6.5
Free T4	EFLM BV database	6.3
Follicle stimulating hormone (FSH)	EFLM BV database	21.2
Human chorionic gonadotropin (hCG)	CLIA	18
Immunoglobulin E (IgE)	CLIA	20
Insulin	EFLM BV database	31.5
Luteinizing hormone (LH)	EFLM BV database	28.4
N-terminal pro B-type natriuretic peptide (NT-ProBNP)	SEQCML BV database	13
Estradiol	EFLM BV database	17.3
Parathyroid hormone	EFLM BV database	20
Procalcitonin	^b^	20.3
Prolactin	EFLM BV database	37.4
PSA, free	EFLM BV database	17.5
PSA, total	EFLM BV database	16.2
Testosterone	EFLM BV database	16.5
Troponin T	EFLM BV database	17.6
Thyroid-stimulating hormone (TSH)	EFLM BV database	24.6
Vitamin B12	CLIA	20

EFLM BV Database, European federation of clinical chemistry and laboratory medicine biological variation database [23]; CLIA, clinical laboratory improvement amendments [25]; RCPA, the royal college of pathologists of Australasia [26]; RiliBAK, German medical association on quality assurance in medical laboratory examinations [27]; SEQCML BV Database, the biological variation database provided by Spanish society of laboratory medicine (SEQCML) analytical quality commission [24]; NCEP, the national cholesterol education program [19], [20], [21]; IFCC Task Force, international federation of clinical chemistry and laboratory medicine task force on implementation of HbA_1c_ standardization [22]; ^a^The TEa for ASO was chosen arbitrarily as data was not available. ^b^Reference [28].

Sigma metric was computed using the TEa, bias, and precision (expressed as coefficient variation, %CV) as the following formula: Sigma=[(%TEa)−|Bias|]/%CV.

### Total allowable error (TEa)

There are multiple sources for the TEa, but none of them comprises all analytes evaluated in the current study.

The hierarchy model proposed in Stockholm in 1999 and Milan in 2014 was followed to choose APS [4]. In this model, the primary choice for the selection of APS is the clinical outcome, but this is unfortunately available for a few analytes. TEa targets were selected for triglyceride [19], total cholesterol [20], high-density lipoprotein cholesterol [20], low-density lipoprotein cholesterol [21], and glycated hemoglobin (HbA_1c_) [22] based on clinical outcome.

The secondary choice was the desirable TEa based on biological variation obtained from EFLM Biological Variation Database [23]. If there was no data in this database, then the biological variation database created by The Spanish Society of Laboratory Medicine Analytical Quality Commission, was updated every two years until 2014, was consulted [24]. If the biological variation targets were not practical (too stringent) or appropriate (too forgiving) for any test, Clinical Laboratory Improvement Amendments (CLIA) [25], The Royal College of Pathologists of Australasia (RCPA) [26], and Guideline of the German Medical Association on Quality Assurance in Medical Laboratory Examinations – Rili-BAEK goals [27] were referenced next.

Finally, for procalcitonin and anti-streptolysin O (ASO), no TEa data were available from the above sources. The TEa for procalcitonin was obtained from a paper based on the biological variation [28], and a TEa of 10 % was arbitrarily preferred for ASO.

The source of TEa preferred for each assay is listed on Table 1.

### Bias

To calculate bias, the EQA data were collected from RIQAS EQA schemes (Monthly clinical chemistry, specific proteins, glycated hemoglobin, lipid, monthly immunoassay, liquid cardiac, and immunoassay specialty) throughout 1-year from 1 March 2019 to 29 February 2020.

The bias of each analyte was estimated by three different approaches. In the first approach, the difference between the laboratory result and the peer group mean value was first calculated for each of the 12 EQA surveys and then the average bias was calculated by the root mean square of the individual bias values.

In the second approach, Passing–Bablok regression analysis [29] was performed using the laboratory results and the peer group mean values obtained from the 12 EQA surveys. The bias was calculated from the regression equation constructed as y=b+ax, where a was an intercept, b was a slope, and x was a value having the concentration at the mean of the IQC sample. In this approach, the bias of each analyte was computed separately for the IQC level 1 and 2.

When using the bias estimation approaches based on the EQA results, if the standard deviation index for an assay was above 3 or below −3 at an EQA event, the related bias value was not used for Sigma metric estimation.

In the final approach, the difference between the laboratory result and the target value of the IQC sample obtained from the control inserts was first calculated for each month over one year, and then the average bias was estimated by the root mean square of the monthly bias values. The IQC samples were obtained from the manufacturer of the reagent (Roche Diagnostics, Manheim, Germany). Similar to the second approach, the bias of each analyte was estimated individually for the IQC level 1 and 2.

### Precision

The precision was determined through the computation of the %CV from the IQC data. The IQC data was collected during the same time frame as the EQA data.

%CV values were calculated monthly, and then the average precision was estimated. The precision values were estimated separately for the both of IQC samples.

### Categorization of Sigma metrics

Sigma metrics computed using bias values obtained from different estimation approaches were entitled following:

1. If Sigma value was estimated using bias values based on the average of multiple bias values from the EQA surveys, it was entitled “EQA Average Sigma”.
2. If Sigma value was computed using bias values from regression analysis of the EQA results, it was entitled “EQA Regression Sigma”.
3. When Sigma metric was calculated using bias values based on the average of the bias values from the IQC studies, it was entitled “IQC Sigma”.

The resulting Sigma values were categorized based on the “Westgard Sigma Rules” diagram [30] following as: category 1 (<3 of sigma), 2 (≥3 and <4), 3 (≥4 and <5), 4 (≥5 and <6), and 5 (≥6). Then, the number of chemistry and immunoassay analytes falling into each Sigma category was separately determined for different bias determination approaches.

Data analyses were performed using MedCalc Statistical Software version 19.1 (MedCalc Software Ltd, Ostend, Belgium) and Excel Office 2019 (Microsoft, USA).

## Results and discussions

Sigma values estimated using different bias determination approaches are presented in Table 2 and 3 for the IQC level 1 and 2, respectively.

**Table 2:** Sigma metrics calculated by three bias estimation approaches at the IQC level 1.

Analyte	%CV (level 1)	Bias values from average of the EQA results, %	Bias values from the regression analysis of the EQA results	Bias values from the IQC results	The “EQA average sigma” values	The “EQA regression sigma” values	The “IQC sigma” values	Sigma category based on “EQA average sigma” values^a^	Sigma category based on “EQA regression sigma” values^a^	Sigma category based on “IQC sigma” values^a^
Albumin	2.58	3.99	1.49	1.37	1.6	2.5	2.6	1	1	1
ALP	2.52	2.15	0.76	2.96	3.9	4.5	3.6	2	3	2
ALT	1.82	1.87	0.76	2.12	7.8	8.4	7.7	5	5	5
Amylase	1.38	2.49	1.36	1.16	7.7	8.6	8.7	5	5	5
Anti-Streptolysin O	3.10	3.88	2.93	3.47	2.0	2.3	2.1	1	1	1
AST	2.02	4.08	2.65	1.06	4.7	5.4	6.2	3	4	5
Bilirubin, direct	2.26	1.78	0.99	2.17	8.1	8.4	7.9	5	5	5
Bilirubin, total	2.18	1.88	1.67	1.79	4.6	4.7	4.7	3	3	3
C reactive protein	2.54	2.23	7.54	3.17	7.0	4.9	6.6	5	3	5
Calcium	1.41	2.13	0.88	1.06	5.6	6.5	6.3	4	5	5
Chloride	2.33	2.00	1.46	2.18	2.6	2.8	2.5	1	1	1
Cholesterol, HDL	2.28	3.52	11.76	2.93	4.2	0.5	4.4	3	1	3
Cholesterol, LDL	2.73	3.15	0.33	3.70	3.2	4.3	3.0	2	3	2
Cholesterol, total	1.95	2.27	0.91	2.90	3.4	4.1	3.1	2	3	2
Creatine kinase	1.49	1.86	2.52	0.63	13.9	13.5	14.8	5	5	5
Creatinine	3.22	2.83	1.09	2.39	1.4	2.0	1.6	1	1	1
D-dimer	2.64	5.58	6.57	3.68	8.5	8.1	9.2	5	5	5
GGT	1.58	3.35	2.04	1.88	9.8	10.7	10.8	5	5	5
Glucose	1.50	1.51	0.02	1.85	3.3	4.3	3.1	2	3	2
Hemoglobin A1c	1.66	1.85	1.00	0.88	3.0	3.6	3.6	2	2	2
Iron	2.28	1.97	0.73	1.58	5.7	6.3	5.9	4	5	4
LDH	1.51	1.68	0.41	2.58	4.0	4.8	3.4	3	3	2
Lipase	2.05	2.68	1.78	1.55	5.6	6.1	6.2	4	5	5
Lithium	3.12	3.18	1.70	3.68	3.8	4.3	3.6	2	3	2
Magnesium	1.88	2.10	0.87	1.13	3.1	3.8	3.6	2	2	2
Phosphorus	1.70	1.84	0.04	1.97	4.6	5.7	4.6	3	4	3
Potassium	1.79	1.37	0.01	0.65	3.7	4.5	4.1	2	3	3
Protein, total	1.46	2.79	1.76	1.79	3.6	4.3	4.3	2	3	3
Rheumatoid factor	2.58	1.84	1.88	2.24	4.5	4.5	4.4	3	3	3
Sodium	1.57	1.46	1.21	0.60	2.3	2.4	2.8	1	1	1
Triglyceride	1.66	2.68	1.36	1.58	7.4	8.2	8.1	5	5	5
Urea	1.91	2.64	0.85	1.76	7.9	8.9	8.4	5	5	5
Uric acid	1.79	2.25	1.29	1.30	5.9	6.4	6.4	4	5	5
25-OH Vitamin D	9.47	11.89	14.64	5.26	0.1	<0	0.2	1	1	1
AFP	2.75	6.35	2.34	2.21	4.1	5.5	5.6	3	4	4
CA 125	2.31	8.17	7.85	4.59	2.5	2.6	4.0	1	1	3
CA 15-3	4.01	6.13	1.20	5.10	4.5	5.7	4.7	3	4	3
CA 19-9	2.75	7.24	1.41	3.82	3.9	6.0	5.1	2	5	4
CEA	2.35	4.32	1.51	1.74	6.9	8.1	8.0	5	5	5
Ferritin	2.97	6.75	10.22	3.25	3.4	2.3	4.6	2	1	3
Folate	7.37	8.45	5.41	6.94	1.5	1.9	1.7	1	1	1
Free T3	2.89	4.26	2.91	2.63	0.8	1.2	1.3	1	1	1
Free T4	3.63	2.81	3.95	2.36	1.0	0.6	1.1	1	1	1
FSH	2.23	6.31	5.23	2.40	6.7	7.2	8.4	5	5	5
hCG	3.33	6.97	7.48	3.90	3.3	3.2	4.2	2	2	3
IgE	2.25	6.27	6.38	3.35	6.1	6.1	7.4	5	5	5
Insulin	2.17	4.13	4.69	2.07	12.6	12.4	13.6	5	5	5
LH	2.09	3.79	2.87	2.70	11.8	12.2	12.3	5	5	5
NT-ProBNP	3.28	3.74	1.50	2.68	2.8	3.5	3.1	1	2	2
Estradiol	3.89	4.24	8.40	6.32	3.4	2.3	2.8	2	1	1
PTH	1.83	8.21	2.93	6.76	6.4	9.3	7.2	5	5	5
Procalcitonin	2.49	3.45	1.26	3.02	6.8	7.6	6.9	5	5	5
Prolactin	2.48	4.54	4.51	1.51	13.2	13.4	14.4	5	5	5
PSA, free	2.50	3.73	4.87	2.40	5.5	5.1	6.0	4	4	5
PSA, total	2.08	5.89	8.62	2.16	5.0	3.6	6.8	4	2	5
Testosterone	1.98	4.40	2.46	4.26	6.1	7.1	6.2	5	5	5
Troponin ths	2.95	6.82	2.69	2.93	3.7	5.1	5.0	2	4	4
TSH	1.91	1.92	0.70	2.99	11.9	12.5	11.3	5	5	5
Vitamin B12	4.38	4.53	3.70	2.76	4.7	4.9	5.1	3	3	4

^a^The resulting Sigma values were categorized based on the “Westgard Sigma Rules” diagram [30] following as: category 1 (<3 of sigma), 2 (≥3 and <4), 3 (≥4 and <5), 4 (≥5 and <6), and 5 (≥6).

**Table 3:** Sigma metrics calculated by three bias estimation approaches at the IQC level 2.

Analyte	%CV (level 2)	Bias values from average of the EQA results, %	Bias values from the regression analysis of the EQA results	Bias values from the IQC results	The “EQA average sigma” values	The “EQA regression sigma” values	The “IQC sigma” values	Sigma category based on “EQA average sigma” values^a^	Sigma category based on “EQA regression sigma” values^a^	Sigma category based on “IQC sigma” values^a^
Albumin	1.61	3.99	1.68	1.25	2.5	3.9	4.2	1	2	3
ALP	2.10	2.15	0.53	6.13	4.7	5.5	2.8	3	4	1
ALT	1.46	1.87	1.16	1.57	9.7	10.2	9.9	5	5	5
Amylase	1.40	2.49	0.22	1.73	7.6	9.2	8.2	5	5	5
Anti-Streptolysin O	2.83	3.88	0.65	2.72	2.2	3.3	2.6	1	2	1
AST	1.60	4.08	3.19	1.98	6.0	6.5	7.3	5	5	5
Bilirubin, direct	1.88	1.78	0.75	1.19	9.7	10.2	10.0	5	5	5
Bilirubin, total	1.84	1.88	0.17	1.83	5.5	6.4	5.5	4	5	4
C reactive protein	1.89	2.23	0.68	4.73	9.4	10.2	8.1	5	5	5
Calcium	1.38	2.13	1.49	1.20	5.7	6.2	6.4	4	5	5
Chloride	1.79	2.00	0.00	1.86	3.4	4.5	3.4	2	3	2
Cholesterol, HDL	1.95	3.52	1.46	4.11	4.9	5.9	4.6	3	4	3
Cholesterol, LDL	2.43	3.15	0.08	2.87	3.6	4.9	3.8	2	3	2
Cholesterol, total	1.46	2.27	0.94	1.08	4.6	5.5	5.4	3	4	4
Creatine kinase	1.44	1.86	0.68	0.78	14.4	15.2	15.1	5	5	5
Creatinine	2.40	2.83	3.40	1.78	1.9	1.7	2.3	1	1	1
D-dimer	1.31	5.58	8.70	1.56	17.1	14.7	20.2	5	5	5
GGT	1.32	3.35	1.63	1.44	11.7	13.0	13.2	5	5	5
Glucose	1.35	1.51	0.62	0.51	3.7	4.4	4.4	2	3	3
Hemoglobin A1c	1.47	1.85	0.66	0.79	3.4	4.3	4.2	2	3	3
Iron	2.00	1.97	1.81	0.68	6.5	6.6	7.2	5	5	5
LDH	1.41	1.68	0.28	2.11	4.3	5.2	4.0	3	4	3
Lipase	1.87	2.68	1.96	2.74	6.2	6.6	6.1	5	5	5
Lithium	1.98	3.18	1.12	1.33	6.0	7.0	6.9	5	5	5
Magnesium	1.73	2.10	1.79	0.88	3.4	3.6	4.1	2	2	3
Phosphorus	1.43	1.84	0.80	2.05	5.5	6.2	5.4	4	5	4
Potassium	1.49	1.37	0.99	0.66	4.5	4.7	4.9	3	3	3
Protein, total	1.29	2.79	2.16	1.34	4.0	4.5	5.2	3	3	4
Rheumatoid factor	1.77	1.84	0.24	1.33	6.6	7.5	6.9	5	5	5
Sodium	1.55	1.46	0.28	0.68	2.3	3.0	2.8	1	2	1
Triglyceride	1.38	2.68	1.37	1.84	8.9	9.9	9.5	5	5	5
Urea	1.78	2.64	0.63	1.86	8.5	9.6	8.9	5	5	5
Uric acid	1.63	2.25	0.02	1.48	6.5	7.8	6.9	5	5	5
25-OH Vitamin D	3.58	4.53	4.66	2.18	0.1	0.3	1.5	1	1	1
AFP	2.78	6.35	7.78	3.61	4.0	3.5	5.0	3	2	4
CA 125	2.25	8.17	8.70	3.03	2.5	2.3	4.8	1	1	3
CA 15-3	3.36	6.13	6.52	3.02	5.3	5.2	6.2	4	4	5
CA 19-9	2.42	7.24	8.62	3.81	4.4	3.8	5.8	3	2	4
CEA	2.09	4.32	4.94	1.88	7.7	7.4	8.9	5	5	5
Ferritin	2.26	6.75	9.18	2.72	4.5	3.4	6.3	3	2	5
Folate	5.67	8.45	5.79	6.03	2.0	2.5	2.4	1	1	1
Free T3	2.11	4.26	3.72	2.89	1.1	1.3	1.7	1	1	1
Free T4	3.69	2.81	1.09	2.72	0.9	1.4	1.0	1	1	1
FSH	2.09	6.31	7.60	1.79	5.6	5.0	7.8	4	4	5
hCG	3.06	6.97	5.39	3.50	3.6	4.1	4.7	2	3	3
IgE	1.99	6.27	7.01	3.30	6.9	6.5	8.4	5	5	5
Insulin	2.05	4.13	4.99	2.07	13.3	12.9	14.3	5	5	5
LH	1.94	3.79	4.21	1.60	12.7	12.5	13.8	5	5	5
NT-ProBNP	2.42	3.74	2.63	2.69	3.8	4.3	4.3	2	3	3
Estradiol	2.51	4.24	1.33	8.44	5.2	6.4	3.5	4	5	2
PTH	1.75	8.21	4.40	5.74	6.7	8.9	8.1	5	5	5
Procalcitonin	2.30	3.45	4.10	3.19	7.3	7.0	7.4	5	5	5
Prolactin	2.38	4.54	4.11	1.22	13.8	13.8	15.2	5	5	5
PSA, free	2.48	3.73	3.69	2.56	5.6	5.6	6.0	4	4	5
PSA, total	2.05	5.89	4.72	1.83	5.0	5.6	7.0	4	4	5
Testosterone	2.68	4.38	2.20	5.90	4.5	5.3	4.0	3	4	3
Troponin ths	1.94	1.92	2.40	2.35	5.1	5.4	7.3	4	4	5
TSH	5.61	11.89	10.47	3.77	11.7	11.5	11.5	5	5	5
Vitamin B12	2.12	6.82	6.15	2.08	6.2	6.5	8.4	5	5	5

^a^The resulting Sigma values were categorized based on the “Westgard Sigma Rules” diagram [30] following as: category 1 (<3 of sigma), 2 (≥3 and <4), 3 (≥4 and <5), 4 (≥5 and <6), and 5 (≥6).

The data used to estimate bias values are provided in the supplementary files. Bias values based on the EQA results are presented monthly in Supplementary Table 1. No bias value was excluded due to the standard deviation index. Bias values estimated by the IQC results are given monthly in Supplementary Tables 2 and 3 for control levels 1 and 2, respectively. In addition,Supplementary Table 4 shows the regression equation derived from the Passing–Bablok regression analysis for each analyte, as well as the target values used to estimate the bias.

For 64 % of chemistry assays at the IQC level 1 and 73 % at level 2, the “EQA Regression Sigma” values were observed to be higher by at least 0.5 when compared to the “EQA Average Sigma” values. Similarly, for 42 % of chemistry assays at the IQC level 1 and 45 % at level 2, the “IQC Sigma” values were found to be higher than the “EQA Average Sigma” values. In addition to this, when comparing with the “IQC Sigma” values, “EQA Regression Sigma” values were encountered to be higher in 9 chemistry assays while the “IQC Sigma” values in 5. These findings demonstrate that the bias estimation based on regression analysis of the EQA results has the potential to yield higher Sigma values for chemistry analytes.

In contrast to chemistry assays, for immunoassay analytes, Sigma values estimated using bias values based on the IQC results were higher than that computed by the other bias estimation approaches. For 65 % of immunoassays analytes at the IQC level 1 and 69 % at level 2, the “IQC Sigma” values were observed to be higher by at least 0.5 compared to the “EQA Average Sigma” values. Likewise, for 42 % of immunoassays analytes at the IQC level 1 and 62 % at level 2, the “IQC Sigma” values were found to be higher than the “EQA Regression Sigma” values. Moreover, there was a difference of at least 0.5 between the “EQA Average Sigma” values and the “EQA Regression Sigma” values in 13 assays at the IQC level 1 and 9 assays at the IQC level 2.

This difference led some assays to be classified into different Sigma categories. Twenty-three analytes (15 chemistry, 8 immunoassays) at IQC level 1 and 21 (14 chemistry, 7 immunoassays) at level 2 were classified into different Sigma categories depending on whether considering the “EQA Average Sigma” or the “EQA Regression Sigma” values. In addition, for 18 analytes (7 chemistry, 11 immunoassays) at IQC level 1 and 20 (8 chemistry, 12 immunoassays) at level 2, the Sigma categories were observed to be different depending on whether the “EQA Average Sigma” or the “IQC Sigma” values were considered. Similarly, if the bias was derived from the regression analysis of bias values from the EQA studies, instead of IQC data, 19 analytes (11 chemistry, 8 immunoassays) at IQC level 1 and 23 (12 chemistry, 11 immunoassays) at level 2 fell into different Sigma categories. For most of the assays with a Sigma value greater than 6, Sigma categories were observed to be unchanged depending on different bias estimation approaches. This is due to Sigma values equal to or greater than 6 are classified in the same category, so that the greater the Sigma value the greater the difference required to produce a change in this Sigma category. For example, although there was a difference of 2.2 units between the “EQA Average Sigma” and the “EQA Regression Sigma” for PTH at IQC level 2, the two fell into the Sigma category 5.

In the present study, Sigma values were separated into categories considering the Westgard Sigma Rules diagram [30]. Therefore, the observed difference in the Sigma category depending on the bias estimation approaches for an assay can alter the QC rules to be chosen, the number of control measurements required per run, and the running frequency of control samples. Using as an example AST, the “IQC Sigma” performance at the IQC level 1 requires only a single control rule, 1_3s_, with 2 control measurements in each run one on each level of control, whereas the “EQA Average Sigma” quality requires 4 rules, 1_3s_/2_2s_/R_4s_/4_1s_, with 4 control measurements in each run or 2 control measurements in each of 2 runs.

In addition to this, the observed variations in Sigma categories will have changed the judgement about the performance of an existing analytical system in the laboratory. When evaluating analytical performance of the chemistry assays by considering Sigma of <3 as unacceptable, albumin, creatinine, sodium, chloride, and ASO was classified in this category at the IQC level 1, regardless of the bias estimation approaches. In addition to these analytes, the “EQA Regression Sigma” value for HDL-cholesterol was observed to be <3. At the IQC level 2, creatinine, sodium, and ASO showed a Sigma performance of <3 considering both the “IQC Sigma” values and the “EQA Average Sigma” values. Moreover, ALP had an “IQC Sigma” value of <3.

At both the IQC level 1 and 2, FT3, FT4, folic acid, and 25-OH vitamin D showed a Sigma performance <3, irrespective of the bias estimation approaches. In addition to these analytes, the “EQA Average Sigma” and “EQA Regression Sigma” value for CA 125 was observed to be <3 at both the IQC level 1 and 2. On the other hand, at the IQC level 1, estradiol had a Sigma performance of <3 taking into consideration the “EQA Regression Sigma” or the “IQC Sigma” value, as well as NT-ProBNP had the “EQA Average Sigma” value of <3, and ferritin had the “EQA Regression Sigma” value of <3.

Bias estimation approaches evaluated here each has an advantage or disadvantage. Ideally, the EQA organizations using commutable control materials with assigned values by reference methods should be preferred to verify the accuracy of laboratory results and their impact on patient samples [31]. However, several EQA providers use commutable or non-commutable control materials but without values assigned by reference methods [31]. On the other hand, there are no reference materials or reference methods for several analytes commonly tested in laboratory medicine. The EQA target values based on peer-group (laboratories using the same method, analytical platform, and reagents) means were used in the present study. In that case, the uncertainty of the peer-group mean value, which depends on the imprecision of data used and the number of laboratories in the peer group, has importance on the observed bias. Although there is a direct relationship between the uncertainty and sample size of the peer group, standardization about how many participants are required in a peer-group is not present. In a previous study [17], a peer group with at least 5 participants was considered adequate for bias estimation. In the present study, the number of laboratories in the peer group was relatively high, with at least 100 participants for immunoassays and 30 for chemistry assays.

For bias estimation from the EQA results, another limitation is that bias estimated in each EQA event is based on a single measurement result. Therefore, bias estimation in this way is susceptible to random errors, especially in case of using bias values from a few EQA studies. Bias estimation on a larger period may help to diminish the effect of possible random errors. In a recent study [15], authors recommended that laboratories calculate Sigma metrics using the data from at least 6 months. On the other hand, when using regression analysis for bias estimation by the EQA results, the sample size becomes a more complicated issue. The number of data pairs recommended for Passing–Bablok regression analysis is 30 [32]. However, it is not easy to achieve this sample size using the EQA surveys. By collecting the data over one year, 12 data points could be obtained for the regression analysis in this study.

In addition to the sample size, the distribution of values is another important point in regression analysis. For example, when the regression analysis was performed using the data points including the concentrations of HDL-cholesterol ranging from 46 to 134 mg/dL, the resulting regression equation yielded a bias of 1.46 % and 11 % for the concentrations of 73.8 and 28.6 mg/dL, respectively. This was attributed to the lack of points with low concentration in the data set used in regression analysis.

Similar to the EQA target values, the IQC target values are not assigned by reference method analysis. In addition to this, although the IQC target values are recommended to be established by the laboratory [33], laboratories commonly rely on the mean values presented by the assay manufacturer in control package inserts. Therefore, the uncertainty of the target values is also an issue for bias estimation based on the IQC data. However, the approach based on the mean of bias values of the IQC results is performed using more data points than those based on the EQA results, which makes bias estimation based on the IQC data more robust to random errors.

There is heterogeneity between previous studies as to bias estimation. The studies evaluating the analytical performance of a new system by Sigma metrics have commonly calculated bias from data based on the method comparison study [7], [8], [9]. On the other hand, bias has been computed using the IQC and EQA results when Sigma metric analysis was performed to plan the IQC studies or assess the analytical performance of an existing system [10], [11], [12], [13], [14], [15].

Tran et al. [17] evaluated Sigma metrics of 20 chemistry analytes on the Beckman Coulter AU680 using the bias estimation approach based on regressing analysis of the EQA results. Similar to the findings in the present study, they computed a Sigma performance of <3 for albumin, creatinine, sodium, and chloride at the IQC level 1. Authors have also reported that calcium, total protein, glucose, and CK had a Sigma value of <3 at the IQC level 1. Moreover, for 6 of the 8 assays at the IQC level 1, the same findings have been reported for the IQC level 2. The difference in Sigma values between the two studies was attributed to the difference in TEa values considered for the assays, except glucose and CK. The reason of low Sigma values for glucose and CK was higher bias values in the cited study. The difference in bias values between the two studies may be partly explained by the difference in the analytical platforms used. Moreover, the distribution of the data used in the regression analysis may have affected the bias values. Unfortunately, the relevant data is not available in the cited study.

In another study, Nar and Emekli [14] estimated Sigma metrics of 18 immunoassay analytes on Cobas e601 analyzer, 17 of which are the same as the present study, to evaluate analytical performance. Authors computed the bias by determining the difference between the target values (mean values) in the control inserts and the observed mean values. Similar to the current study, they have reported a Sigma value of <3 for FT4. Moreover, unlike the findings from this study, Sigma value of <3 have been also reported for AFP. On the other hand, they have estimated a Sigma of >6 for folic acid which was of Sigma of <3 in the present study. A similar Sigma performance should be achieved when laboratories use the same analytical platform and reagent formulation. Therefore, in the case of poor individual-site Sigma performance, local issues that influence analytic performance should be targeted. These factors may be instrumentation malfunctions, reagent issues, sample handling, or possible operator-related errors.

Kumar and Mohan [12] assessed the Sigma performance of 16 chemistry assays on VITROS 4600 using the average bias from monthly bias values based on the EQA results. The authors reported a Sigma performance similar to that in this study for sodium and albumin, but the Sigma values for creatinine were higher. Despite similar bias and imprecision values for creatinine, the observed difference in Sigma values was due to the difference in TEa values considered in the studies. Authors have selected a TEa of 15 % based on state-of-art whereas a TEa of 7.5 % based on biological variation was preferred in the present study. They have also reported Sigma values of <3 for urea using a TEa of 9 % based on state-of-art while Sigma value of >6 was determined using a TEa of 17.6 % based on biological variation in the current study.

The influence of this heterogeneity in bias estimation on the resulting Sigma metrics is investigated in a few studies [10, 15]. In a study by Guo et al. [10], Sigma metrics for 10 chemistry assays have been estimated using two approaches in calculation of bias and imprecision, one of which was based on only the IQC results and another one was only the EQA results. In the approach based on the IQC results, bias had been estimated by comparison with peer group from the IQC program. Interestingly, in the approach based on the EQA results, authors used the EQA samples to estimate imprecision following a precision study over relatively short-term period along with bias estimation. Similar to the findings from the present study, they stated that the sigma levels from the two methods were significantly different, which could affect outcomes for the IQC rule selection.

In another more recent study [15], Wauthier et al. compared sigma metrics for 20 analytes computed using two bias estimation methods based on the comparing peer groups from the EQA or the IQC program. In contrast to the findings from the present study, they concluded that the source of bias was less decisive for Sigma metrics. When evaluating the impact of bias estimation approach on sigma metrics, authors categorized sigma metrics as <3, 3–6, and >6. In other words, the differences in sigma metric between 3 and 6, which are of importance in selecting the IQC rules and frequency, have not been assessed. This has potential to hindrance the identifying the effect of bias estimation methods on resulting Sigma metrics.

Furthermore, alongside the conventional approach, Coskun et al. introduced a new model to calculate Sigma metrics in laboratory medicine [34]. In this novel model, Sigma metric is determined by incorporating the analytical imprecision and tolerance limits. Unlike the traditional method, bias is not directly included in the equation of the Sigma metric. Instead, the Sigma metric value is converted into defects per million opportunities, assuming the presence of a 1.5 standard deviation bias. As a result, the proposed model facilitates the evaluation of analytical methods using Sigma metrics. However, this model exclusively considers tolerance limits based on biological variation, disregarding other approaches for defining tolerance limits. This limitation has the potential to restrict the application of the new model to laboratory testing processes, as biological variation data may not be available for all measurands or may not be relevant for certain measurands. Additionally, it should be noted that this model does not provide a methodology for selecting or designing the IQC rules [35]. Therefore, in the present study, only the traditional formula was utilized to estimate Sigma metrics.

This study has some limitations. At first, bias should be ideally estimated by comparison to reference methods [16], but the bias estimation could be only performed using the IQC and EQA data in the present study. Therefore, the bias values reported here should be considered relative rather than absolute. The selection of TEa goals from multiple sources was another limitation in the present study. If one chooses a different TEa value from that used in the present study, different Sigma metrics can be obtained. Unfortunately, there are no universally accepted TEa values to be used for Sigma calculation. The IFCC working group on HbA_1c_ standardization recommends [22] a specific TEa value for Sigma metric calculation of HbA_1c_. They have defined the TEa value by taking into consideration the analytical performance of existing methods in the field. Like HbA_1c_, establishing specific TEa values for Sigma computation can help overcome the challenge of deciding on the appropriate TEa source.

In conclusion, Sigma metrics may differ depending on whether bias is derived by the averaging or regression analysis of the EQA results or obtained from the IQC results. This should be taken into consideration when using Six Sigma to evaluate analytical performance or design the IQC events.

## Supplementary Material

Supplementary Material

## References

[1] Forsman RW (1996). Why is the laboratory an afterthought for managed care organizations?. Clin Chem.

[2] Westgard S, Bayat H, Westgard JO (2018). Analytical sigma metrics: a review of Six Sigma implementation tools for medical laboratories. Biochem Med.

[3] Westgard JO (2006). Six sigma quality design and control.

[4] Sandberg S, Fraser CG, Horvath AR, Jansen R, Jones G, Oosterhuis W (2015). Defining analytical performance specifications: consensus statement from the 1st strategic conference of the European federation of clinical chemistry and laboratory medicine. Clin Chem Lab Med.

[5] Hens K, Berth M, Armbruster D, Westgard S (2014). Sigma metrics used to assess analytical quality of clinical chemistry assays: importance of the allowable total error (TEa) target. Clin Chem Lab Med.

[6] Xia Y, Xue H, Yan C, Li B, Zhang S, Li M (2018). Risk analysis and assessment based on Sigma metrics and intended use. Biochem Med.

[7] Westgard S, Petrides V, Schneider S, Berman M, Herzogenrath J, Orzechowski A (2017). Assessing precision, bias and sigma-metrics of 53 measurands of the Alinity ci system. Clin Biochem.

[8] Taher J, Cosme J, Renley BA, Daghfal DJ, Yip PM (2019). A novel Sigma metric encompasses global multi-site performance of 18 assays on the Abbott Alinity system. Clin Biochem.

[9] Fasano T, Bedini JL, Fle PA, Jlaiel M, Hubbert K, Datta H (2019). Multi-site performance evaluation and Sigma metrics of 20 assays on the Atellica chemistry and immunoassay analyzers. Clin Chem Lab Med.

[10] Guo X, Zhang T, Gao X, Li P, You T, Wu Q (2018). Sigma metrics for assessing the analytical quality of clinical chemistry assays: a comparison of two approaches. Biochem Med.

[11] Yang F, Wang W, Liu Q, Wang X, Bian G, Teng S (2020). The application of Six Sigma to perform quality analyses of plasma proteins. Ann Clin Biochem.

[12] Kumar BV, Mohan T (2018). Sigma metrics as a tool for evaluating the performance of internal quality control in a clinical chemistry laboratory. J Lab Physicians.

[13] Zhou B, Wu Y, He H, Li C, Tan L, Cao Y (2020). Practical application of Six Sigma management in analytical biochemistry processes in clinical settings. J Clin Lab Anal.

[14] Nar R, Emekli DI (2017). The evaluation of analytical performance of immunoassay tests by using Six-sigma method. J Med Biochem.

[15] Wauthier L, Chiaro LD, Favresse J (2022). Sigma metrics in laboratory medicine: a call for harmonization. Clin Chim Acta.

[16] Clinical and Laboratory Standards Institute (2013). Measurement procedure comparison and bias estimation using patient samples. EP09c.

[17] Tran MT, Hoang K, Greaves RF (2016). Practical application of biological variation and Sigma metrics quality models to evaluate 20 chemistry analytes on the Beckman Coulter AU680. Clin Biochem.

[18] Feldhammer M, Brown M, Colby J, Bryksin J, Milstid B, Nichols JH (2021). A Survey of Sigma metrics across three academic medical centers. J Appl Lab Med.

[19] Stein EA, Myers GL (1995). National cholesterol education program recommendations for triglyceride measurement: executive summary. The national cholesterol education program working group on lipoprotein measurement. Clin Chem.

[20] Warnick GR, Wood PD (1995). National cholesterol education program recommendations for measurement of high-density lipoprotein cholesterol: executive summary. The national cholesterol education program working group on lipoprotein measurement. Clin Chem.

[21] Bachorik PS, Ross JW (1995). National cholesterol education program recommendations for measurement of low-density lipoprotein cholesterol: executive summary. The national cholesterol education program working group on lipoprotein measurement. Clin Chem.

[22] Weykamp C, John G, Gillery P, English E, Ji L, Lenters-Westra E (2015). Investigation of 2 models to set and evaluate quality targets for HbA_1c_: biological variation and sigma-metrics. Clin Chem.

[23] Aarsand AK, Fernandez-Calle P, Webster C, Coskun A, Gonzales-Lao E, Diaz-Garzon J The EFLM biological variation database. ..

[24] Westgard QC Biologic variation database, the 2014 update. ..

[25] Department of Health and Human Services Clinical Laboratory Improvement Amendments of 1988 (CLIA) proficiency testing regulations related to analytes and acceptable performance. ..

[26] The Royal College of Pathologists of Australasia. Allowable limits of performance – RCPAQAP. ..

[27] German Medical Association (2015). Revision of the “Guideline of the German medical association on quality assurance in medical laboratory examinations – Rili-BAEK” (unauthorized translation). J Lab Med.

[28] Barassi A, Pallotti F, Melzi d’Eril G (2004). Biological variation of procalcitonin in healthy individuals. Clin Chem.

[29] Passing H, Bablok W (1983). A new biometrical procedure for testing the equality of measurements from two different analytical methods. Application of linear regression procedures for method comparison studies in clinical chemistry, part I. J Clin Chem Clin Biochem.

[30] Westgard JO, Westgard SA (2016). Quality control review: implementing a scientifically based quality control system. Ann Clin Biochem.

[31] Ricós C, Fernández-Calle P, Perich C, Sandberg S (2022). External quality control in laboratory medicine. Progresses and future. Adv Lab Med.

[32] Bablok W, Passing H (1985). Application of statistical procedures in analytical instrument testing. J Automat Chem.

[33] Clinical and Laboratory Standards Institute (2016). Statistical quality control for quantitative measurement procedures: principles and definitions.

[34] Coskun A, Serteser M, Kilercik M, Aksungar F, Unsal I (2015). A new approach to calculating the Sigma Metric in clinical laboratories. Accred Qual Assur.

[35] Westgard S, Bayat H, Westgard JO (2019). Mistaken assumptions drive new Six Sigma model off the road. Biochem Med.

