# Supplementary material for: Comparison of Sigma metrics computed by three bias estimation approaches for 33 chemistry and 26 immunoassay analytes
Source: Adv Lab Med. 2023 Jul 4;4(3):236–45. doi: 10.1515/almed-2022-0095 (PMC10756147; doi:10.1515/almed-2022-0095)
Supplement: Supplementary file 1 — Supplementary Material [file j_almed-2022-0095_suppl_001.docx]

Supplementary Table 1: Monthly bias values obtained from the EQA schemes

| Analyte | %Bias Values | | | | | | | | | | | |
| --- | --- | --- | --- | --- | --- | --- | --- | --- | --- | --- | --- | --- |
|  | March  2019 | April  2019 | May  2019 | June  2019 | July  2019 | August  2019 | September  2019 | October  2019 | November  2019 | December  2019 | January  2020 | February  2020 |
| Albumin | 1.6 | 9.4 | 1.9 | 1.7 | 1.4 | -2.2 | 4.4 | 12.2 | -0.6 | -5.4 | 4.4 | -2.7 |
| ALP | -0.4 | 10.3 | 1.3 | 1.8 | 0.7 | -6.4 | -1.1 | -0.1 | -0.1 | -1.5 | 1.9 | 0.2 |
| ALT | 0.7 | -1.7 | 0 | -0.3 | 1.7 | -1.4 | 3.6 | 0.5 | 2.1 | -2.6 | 2.3 | 5.5 |
| Amylase | -0.2 | -5.4 | 1.5 | 2.1 | 0.2 | -6.6 | 0.6 | -1.8 | -1.8 | -2.7 | 0.4 | -6.6 |
| Anti-Streptolysin O | 0.9 | -2 | -9.1 | -2.3 | 7.2 | 4 | * | 1.3 | 4 | 3 | 6 | 2.9 |
| AST | 0.5 | 5.5 | 3.8 | 5.6 | 3 | -5.7 | 3.5 | 2 | 0.9 | -3.3 | 4.5 | 10.7 |
| Bilirubin, Direct | -3.2 | 4.3 | 0.9 | -1.1 | -1.8 | -3.9 | 1 | -0.8 | -0.5 | -1.8 | -0.4 | -1.7 |
| Bilirubin, Total | -2.2 | -1.6 | 1.1 | -1.3 | -0.6 | -2.7 | 2.2 | -0.2 | 2.8 | -3.4 | 1.5 | 3 |
| C Reactive Protein | 1.6 | -4.1 | 0.2 | 3.3 | 2.4 | 3.5 | * | 2.8 | 0.2 | 1.7 | -2.4 | 2.3 |
| Calcium | 2.6 | 1.7 | 2.1 | 0.9 | 1.4 | -6 | -3 | -0.7 | 1.6 | -4.4 | -1 | 0.2 |
| Chloride | 0.2 | -1.3 | -2 | -3 | 0.3 | -6.5 | 0.7 | -1 | -3.2 | 2 | 0.6 | 3.2 |
| Cholesterol, HDL | -4.4 | -2.3 | -0.2 | 0 | -4.8 | -8.4 | -2.3 | 6.7 | 4.7 | -4.1 | 2.8 | 1.5 |
| Cholesterol, LDL | 7 | 6.7 | 5.2 | * | 0 | -3.1 | 2.6 | 0.9 | 2.3 | 3.8 | -0.7 | -2.3 |
| Cholesterol, Total | 1.7 | 2.5 | 4.5 | 4.5 | 3.6 | -3.8 | 0.4 | 0.7 | 0.5 | -3 | -0.5 | 1.5 |
| Creatin Kinase | -1 | -1.1 | -2.4 | -2.4 | 0.2 | -3.4 | 2.3 | 0.4 | -1.9 | -2.2 | 3.3 | 1.7 |
| Creatinine | 0.2 | -3.1 | 4.1 | 5.1 | 2.6 | -0.9 | 0.7 | 4.1 | 0.8 | -9.6 | 1 | 1.7 |
| D-Dimer | -3.3 | 8.79 | 11.7 | 14.4 | 3.4 | -1.5 | -5 | -3.7 | 5.8 | 3.1 | 2.3 | 4 |
| GGT | 4.5 | 8.2 | 3.1 | 5 | 1.5 | -3.6 | 1.5 | 1 | 3.2 | -0.9 | 1.7 | 6 |
| Glucose | 1.4 | 2.2 | 1.6 | -0.1 | -0.5 | -5.6 | 0.3 | -0.2 | 0.7 | -3 | -0.2 | 2.3 |
| Hemoglobin A1c | -0.2 | 2.8 | 2.3 | -0.6 | 3.7 | -8 | 0.2 | -0.1 | 0.7 | 0.7 | -1.1 | 1.8 |
| Iron | -1.1 | -0.9 | -0.1 | 1.9 | -2 | -0.3 | 4.5 | 2.5 | 0.7 | -3 | 1.5 | -5.1 |
| LDH | -0.8 | 2.1 | 1.8 | 2.7 | 0.1 | -4.7 | -0.2 | 0.4 | 1.4 | -4.1 | 0 | 1.9 |
| Lipase | -1.7 | -10.4 | 1.6 | -1.1 | -2.4 | -3.2 | -1.1 | -3.5 | 2.9 | -1.5 | -2.7 | 0 |
| Lithium | 5.7 | 0.6 | -1.6 | 2.6 | -1.1 | -0.4 | 2.9 | 2.4 | -11.6 | -1.4 | 1.6 | 6.3 |
| Magnesium | 0.1 | 2.1 | 2.9 | 2.6 | 2.3 | -2.9 | -0.4 | 2.2 | 0.8 | -4.7 | 0.6 | 3.6 |
| Phosphorus | 0.6 | -3.2 | -2.5 | 0.1 | -0.1 | -2.4 | 1 | -0.3 | 2.6 | -1.8 | 3.9 | -3.6 |
| Potassium | -0.4 | -1.4 | 0.8 | 0.6 | 1.9 | -3.4 | 1.5 | -1.1 | -0.4 | 0 | 1.1 | 3.8 |
| Protein, Total | -0.4 | -1.6 | 5.2 | 0.4 | 2 | -7.4 | 2.4 | 1.4 | 2.4 | -1.3 | 3.7 | 5.3 |
| Rheumatoid factor | 2.7 | -1.5 | -4.8 | 1.1 | 1.4 | -3.8 | * | -0.8 | 0.2 | -1.3 | -2.4 | 0.2 |
| Sodium | 0.6 | 1.4 | 2.2 | 0.6 | 1.5 | -3.1 | 1.6 | -0.3 | -0.7 | -0.1 | 1.7 | 3.7 |
| Triglyceride | -0.4 | 2.7 | 1.3 | 3.5 | 0.4 | -6.6 | -1.7 | 3.1 | 5.2 | -2.8 | 1.9 | 2.5 |
| Urea | 4.7 | 5.4 | 3.9 | 1.7 | -0.6 | -1.2 | 2.5 | -1.2 | -1.3 | -4.5 | -1.5 | -3.2 |
| Uric Acid | -2.6 | 1 | 3.8 | 2 | 0.9 | -5.2 | -0.5 | -2.6 | -3.8 | -3.1 | -0.2 | 1.3 |
| 25-OH Vitamin D | 23.1 | 15.1 | 15.8 | 21.6 | 2.2 | -4.9 | 10.7 | 8.7 | 21.4 | 9.7 | 4.9 | 4.6 |
| AFP | 7.1 | 8.8 | 7.3 | 9.5 | 11.8 | 1.4 | 6.4 | 10.5 | 1.7 | 1.1 | 8.4 | 2.2 |
| CA 125 | 8.9 | 13.9 | 7.7 | 9.6 | 8.8 | 0.6 | 6.4 | 11 | 10.1 | 0.7 | 13.3 | -7 |
| CA 15-3 | 10.2 | 9.2 | 6.2 | 5.3 | 14 | 0.2 | 3.8 | 6.3 | 3.5 | -0.1 | 3.8 | -11 |
| CA 19-9 | 0.9 | 9.1 | 2.6 | 4.4 | 8.1 | 2.1 | 7.2 | 6.9 | 7.5 | 10.3 | 17 | 10.8 |
| CEA | 6.6 | 7 | 6.3 | 5 | 5.6 | -2.5 | 2.4 | 4 | 2.1 | -1.8 | 8.2 | 0.3 |
| Ferritin | 7.6 | 15.5 | 1.5 | 10.7 | 10.5 | -7.4 | 2.8 | 11.5 | -5.4 | 2.1 | 2.5 | 3.5 |
| Folate | 6.2 | 2.8 | 4.1 | -2.1 | 3.5 | 10.5 | -9.3 | -1.2 | 25.1 | 9.1 | 10.3 | 17.2 |
| Free T3 | 3 | 9.8 | 2.5 | 5 | -0.1 | 1.1 | 4.8 | 6.5 | 5.3 | 0.3 | 3.9 | -8.8 |
| Free T4 | -0.1 | 5 | 5 | 4.2 | -3.3 | -3.8 | 1.4 | 2.8 | 1.8 | -0.6 | 5.3 | -0.4 |
| FSH | 7.3 | 11 | 10.2 | 10.5 | 9.7 | 3.9 | 5.6 | 5.4 | 2.3 | 2.7 | 5.8 | -1.3 |
| hCG | 17 | 8.2 | 10.1 | 5.6 | 4.5 | 13.6 | 8.6 | 4 | 0.1 | 5.2 | 3.2 | 3.5 |
| IgE | 1.4 | 8.5 | 11 | 6.5 | 8.6 | 1.2 | 8.6 | 8.6 | 8.1 | 4.7 | 3.6 | 4.4 |
| Insulin | 6.4 | 3.7 | 5.3 | 1.5 | 6.5 | -1.6 | 4.9 | 5.1 | -2.5 | 2.9 | 6.5 | 2.7 |
| LH | 7.5 | 5.6 | 1.6 | 4.5 | 3.9 | -5.2 | 0.5 | 3.7 | -4.6 | -1.6 | 0.5 | 6.3 |
| NT-ProBNP | -2.4 | 19.3 | 3.9 | -0.3 | 3.08 | 4.8 | 2.3 | 0.5 | -2.2 | -0.38 | -3 | 2.7 |
| Estradiol | -1 | 2.9 | -0.3 | 2.3 | -0.6 | 16.6 | 5.3 | 7.5 | 6.9 | -0.8 | 3 | 3.7 |
| PTH | -4.7 | 8 | -17.4 | -15 | 10.9 | -1.9 | 14 | 5.8 | -16.6 | 0.1 | -0.1 | 4 |
| Procalcitonin | 4.5 | -0.2 | 5.3 | 6.1 | 3.4 | 3 | 5.3 | 5 | 2.2 | -1.5 | 2.3 | 2.6 |
| Prolactin | 8.1 | 8.4 | 9.6 | 5.9 | 6.5 | 2.4 | 1.8 | 2 | 1.7 | 0.3 | 4.9 | 2.9 |
| PSA, Free | 1.8 | 3.7 | 5.6 | 5.2 | 3.2 | -0.6 | 4.4 | 1.6 | 7.2 | 1 | 3.6 | 6.9 |
| PSA, Total | 8.5 | 4.4 | 10 | 8 | 8.7 | 1.8 | 5.4 | 7.9 | 6.2 | 4.7 | 1.5 | 3.6 |
| Testosterone | -5.1 | 10.1 | -0.1 |  | 0.5 | 0.3 | 4 | 8.4 | 12.4 | 2.1 | 2.7 | 2.5 |
| Troponin Ths | -3.1 | -9.9 | -6.5 | 3.2 | -15.5 | 1.8 | -5.1 | 7.9 | 6.8 | -9.81 | 6 | 6.2 |
| TSH | 1.2 | 2.9 | 1.6 | 2.4 | 1.5 | 0.3 | 2.3 | 0 | -1.2 | -1 | 5.9 | 2.7 |
| Vitamin B12 | 5.6 | 6.5 | 6.2 | 6.9 | 0.8 | -2.5 | -2.6 | -3 | 10.3 | -1.6 | 3.1 | 5.3 |

* No data were available for the relevant EQA survey.

Supplementary Table 2: Monthly bias values estimated using the IQC level 1 results

| Analyte | Target Value  (IQC Level 1) | %Bias Values | | | | | | | | | | | |
| --- | --- | --- | --- | --- | --- | --- | --- | --- | --- | --- | --- | --- | --- |
|  |  | March  2019 | April  2019 | May  2019 | June  2019 | July  2019 | August  2019 | September  2019 | October  2019 | November  2019 | December  2019 | January  2020 | February  2020 |
| Albumin | 32.7 | -0.23 | -0.36 | 0.3 | 0.49 | 2.6 | -2.2 | 1.53 | 4.62 | 1.36 | -1.14 | 1.49 | -0.07 |
| ALP | 94.9 | -1.1 | 0.47 | -0.02 | 1.35 | -5.48 | -5.27 | -4.14 | -3.03 | -1.38 | -3.32 | -3.99 | -5.97 |
| ALT | 48 | -3.87 | -2.56 | -2.98 | -1.75 | 0.87 | -1.98 | -1.3 | -2.22 | -1.75 | -1.94 | 1.58 | 2.58 |
| Amylase | 76.3 | -1.22 | -0.22 | -0.39 | 0.61 | -1.11 | -1.1 | -2.09 | -1.43 | -1.09 | -0.49 | -2.12 | -2.02 |
| Anti-Streptolysin O | 122 | 2.21 | -1.3 | -1.85 | -0.82 | 4.66 | 5.05 | 8.49 | 4.89 | 5.25 | 5.82 | 1.14 | 0.2 |
| AST | 46.4 | -2.07 | 0.98 | 0.71 | 0.79 | 1.83 | -0.66 | -0.15 | 0.08 | 0.21 | -0.87 | 0.88 | 3.45 |
| Bilirubin, direct | 0.994 | -1.95 | -1.25 | -2.05 | -1.49 | -2.14 | -3.26 | -2.99 | -2.53 | -2.17 | -2.76 | -2.03 | -1.38 |
| Bilirubin, Total | 1.05 | -2.03 | -1.55 | -2.94 | 0.14 | 2.27 | 0.06 | 1.66 | 1.18 | 1.96 | 1.38 | 3.34 | 2.96 |
| C Reactive Protein | 7.96 | -0.47 | -0.88 | 2.56 | 6.89 | 2.9 | 3.64 | 2.75 | 3.69 | 2.67 | 6.06 | 3.6 | 1.87 |
| Calcium | 8.9 | 1.68 | 0.89 | -0.84 | 0.13 | 0.43 | -2.52 | -2.84 | -1.31 | 0.24 | 1.45 | -0.03 | -0.32 |
| Chloride | 76.5 | 4.07 | 1.12 | -0.48 | -0.04 | 8.76 | -3.55 | -1.92 | 0.16 | -0.45 | 1.14 | -3.02 | -1.46 |
| Cholesterol, HDL | 28.6 | -3.43 | -5.69 | -0.18 | 0.57 | -0.35 | -3.67 | -2.95 | 3.99 | 4.85 | 5.31 | 2.6 | 1.53 |
| Cholesterol, LDL | 56.1 | 1.69 | 2.67 | 8.19 | 9.51 | 2.45 | -2.32 | 2.02 | 1.93 | 2.96 | 3.75 | -3.13 | -3.82 |
| Cholesterol, Total | 90.9 | 0.48 | 1.48 | 3.17 | 4.22 | 4.08 | 2.58 | 3.27 | 1.99 | 2.94 | 8.29 | 1.42 | 0.93 |
| Creatin Kinase | 155 | -0.22 | -0.73 | -0.36 | 0.19 | 0.44 | -0.15 | 0.46 | 1.31 | 0.51 | -0.98 | -0.61 | -1.54 |
| Creatinine | 1.07 | 2.1 | 2.14 | 4.66 | 4.65 | -0.02 | 0.29 | 1.75 | 4.13 | 2.88 | 3 | 2.55 | -0.56 |
| D-Dimer | 0.82 | -1.59 | -1.71 | -2.83 | -7.97 | -5.48 | -6.63 | -5.46 | -5.03 | 0.88 | 0.65 | -2.51 | -3.37 |
| GGT | 53.3 | 1.63 | 1.92 | 2.1 | 3.08 | 2.5 | 1.5 | 0.36 | 0.7 | 1.54 | -5.46 | -0.16 | -1.58 |
| Glucose | 100 | 2.41 | 2.66 | 2 | 2.32 | 1.46 | 0.68 | 1.16 | 1.65 | 2.82 | 1.77 | 2.05 | 1.26 |
| Hemoglobin A1c | 5.7 | -0.6 | -0.94 | 0.24 | -0.96 | -2.86 | -0.76 | -0.8 | -1.25 | -0.98 | -0.54 | -0.13 | 0.49 |
| Iron | 105 | 1.44 | 1.02 | 1.81 | 2.74 | 1.33 | 2.41 | 2.75 | 0.15 | 2.02 | 1.96 | 1.2 | -0.07 |
| LDH | 170 | -0.41 | 1.47 | 1.66 | 2.44 | 2.5 | 2.92 | 3.57 | 3.84 | 3.92 | 1.94 | 3.8 | 2.5 |
| Lipase | 45.6 | -0.75 | -1.54 | -1.6 | -1.85 | -2.18 | -0.45 | -0.46 | -1.32 | 1.05 | -6.51 | -0.37 | -0.49 |
| Lithium | 0.9 | 6.3 | 3.89 | 3.06 | 5.74 | 0.88 | 2.92 | 3.2 | 0.91 | 1.13 | 5.52 | 5.37 | 5.2 |
| Magnesium | 1.98 | -1.65 | -1.85 | -1.67 | -0.68 | -0.08 | 0.55 | -0.84 | -0.85 | 0.32 | 2.26 | -1.77 | -1.09 |
| Phosphorus | 4.15 | -2.03 | -2.03 | -2.05 | 0.32 | 1.28 | 1.93 | 2.29 | 1.61 | 3.03 | 1.94 | 2.68 | 2.39 |
| Potassium | 3.66 | 0.89 | 1.2 | 0.63 | 1.78 | 0.14 | 0.84 | 0.67 | 0.36 | 0.34 | -0.52 | 0.22 | -0.17 |
| Protein, Total | 49.3 | -1.18 | -1.21 | 0.96 | 2.36 | 1.27 | -1.39 | 0.98 | 1.97 | 2.89 | 2.51 | 2.25 | 2.47 |
| Rheumatoid factor | 20.5 | 1.9 | 4.04 | 4.59 | 4.48 | 2.18 | 0.51 | -0.61 | 2.52 | 4.02 |  | -0.22 | 1.78 |
| Sodium | 113 | -0.1 | 0 | -0.28 | 0.19 | 0.8 | 1.26 | 1.29 | 1.01 | 1.51 | -0.07 | 0.25 | -0.45 |
| Triglyceride | 121 | 0.38 | 0.95 | 0.97 | 2.8 | 2.9 | -1.1 | -0.79 | 2.22 | 2.86 | 1.14 | 1.42 | 1.41 |
| Urea | 39.3 | 2.3 | 1 | -0.28 | 0.27 | 1.73 | 3.41 | 2.73 | 2.57 | 1.61 | -0.23 | -1.87 | -3.15 |
| Uric Acid | 4.6 | -0.56 | -0.35 | 2.03 | 3.76 | 2.03 | 0.74 | 0.02 | -2.08 | -1.37 | 0.37 | -0.93 | -1.34 |
| 25-OH Vitamin D | 12.92 | -10.36 | -12.45 | -3.24 | -1.28 | -5.01 | -7.83 | -6.86 | -8.92 | -3.12 | -1.48 | -2.07 | 0.45 |
| AFP | 12 | 0.87 | 2.05 | 2 | 3.83 | 3.9 | 2.2 | 3.6 | 1.05 | -3.42 | -1.15 | -0.39 | 2.02 |
| CA 125 | 33.46 | 6 | 6.31 | -3.07 | -3.88 | -4.82 | -6.95 | -5.99 | -5 | -2.42 | -7.2 | -2.48 | -0.9 |
| CA 15-3 | 20.81 | 1.5 | -3.34 | -9.02 | -9.27 | -7.2 | -9.54 | -12.51 | 0.55 | -0.04 | 2.58 | -2.66 | 2.93 |
| CA 19-9 | 25.05 | 0.89 | 0.95 | -2.03 | 1.29 | 5.91 | 10.87 | 11.88 | 2.87 | 3.28 | 3.36 | 1.11 | 1.35 |
| CEA | 5.17 | -0.58 | -0.51 | -1.7 | -0.91 | -0.21 | -1.59 | 2.68 | 1.05 | -1.15 | -4.55 | -2.67 | -3.24 |
| Ferritin | 25.37 | -0.91 | -1.04 | -6.17 | 0.81 | 0.58 | -6.08 | -5.71 | -3.34 | -4.84 | -7.84 | -0.47 | 1.26 |
| Folate | 4.598 | 3.7 | 15.69 | 4.18 | 12.85 | 14.45 | 7.81 | -0.14 | 3.62 | 7.82 | 6.72 | -5.22 | 1.07 |
| Free T3 | 5.5 | -2.57 | 4.55 | 2.51 | 4.8 | 2.99 | 4.03 | -0.95 | 1.11 | 3.12 | 1.82 | 0.63 | 2.53 |
| Free T4 | 1.18 | -7.45 | -2.86 | -0.75 | -0.92 | -3.13 | -1.71 | -2.18 | -0.13 | -0.56 | 3.02 | 3.06 | -2.52 |
| FSH | 18.51 | 1.43 | 4.28 | 1.18 | 1.22 | 1.56 | -0.26 | -2.06 | -2.8 | -4.48 | -3.33 | 2.46 | -3.77 |
| hCG | 5.11 | 6 | 2.97 | -0.61 | 0.6 | -1.29 | 5.83 | -3.89 | -7.43 | -7.48 | -4.25 | -4.76 | 1.68 |
| IgE | 123.78 | -10.52 | 0.23 | 0.74 | 1.01 | 0.07 | -0.68 | 5.26 | 3.46 | 5.6 | 4.54 | 6.36 | -1.76 |
| Insulin | 24.46 | 2.14 | -1.46 | -3.8 | -4.01 | -2.13 | -0.11 | -0.4 | -4.11 | -3.21 | -1.27 | 2.03 | 0.17 |
| LH | 9.77 | -2.75 | 1.53 | -0.18 | 0.46 | -0.35 | -4.72 | 3.35 | 3.92 | 3.14 | 3.17 | 6.99 | 1.85 |
| NT-ProBNP | 134 | 5.87 | 1.68 | -2.41 | -0.93 | 5.57 | 4.4 | 4.42 | -1.42 | -0.03 | -2.18 | -2.3 | 0.89 |
| Estradiol | 105 | -11.82 | -5.11 | -9.31 | -11.81 | -9.72 | -4.02 | -4.57 | -1.35 | -1.68 | -4.94 | -10.2 | -1.34 |
| PTH | 51.6 | 7.32 | 6.74 | 7.45 | 5.09 | 7.71 | 10.11 | 9 | 6.42 | 5.31 | -13.58 | 1.62 | 0.77 |
| Procalcitonin | 0.47 | 4.35 | -0.27 | -1.6 | 0.91 | 1.31 | -2.74 | -5.9 | -7.95 | -1.5 | 1 | 2.52 | 6.15 |
| Prolactin | 223.24 | -1.5 | 0.33 | 0.07 | 0.48 | -0.67 | -2.85 | -2.42 | -2.48 | -3.11 | -0.61 | 2.41 | -1.22 |
| PSA, Free | 0.85 | -5.12 | -4.24 | -0.62 | 0.09 | -1.84 | -2.04 | -0.1 | -0.5 | -1.07 | -8.49 | 2.88 | 1.76 |
| PSA, Total | 3.93 | -2.6 | -2.58 | 2.51 | 0.47 | -2.27 | -2.27 | -1.22 | -0.52 | 0.91 | -3.88 | -3.58 | -3.05 |
| Testosterone | 5.8 | -14.04 | 4.36 | 5.97 | -1.5 | 0.44 | 0.7 | -0.55 | 4.68 | 6.59 | 5.24 | -2.9 | -4.15 |
| Troponin Ths | 28.35 | 3.1 | 3.02 | 5.14 | 5.25 | 4.31 | 1.61 | 0.55 | 1.49 | 0.41 | 2.71 | 4.57 | 2.97 |
| TSH | 1.38 | -9.72 | 2.33 | 0.96 | 3.17 | 2.36 | 4.44 | -2.32 | -3.8 | -1.33 | -2.8 | 2.17 | -0.5 |
| Vitamin B12 | 512.01 | -3.62 | 3.9 | -1.65 | 0.85 | 1.56 | 2.01 | -3.09 | -3.56 | -4.26 | -4.51 | -0.87 | 3.18 |

Supplementary Table 3: Monthly bias values estimated using the IQC level 2 results

| Analyte | Target Value (IQC Level 2) | %Bias Values | | | | | | | | | | | |
| --- | --- | --- | --- | --- | --- | --- | --- | --- | --- | --- | --- | --- | --- |
|  |  | March  2019 | April  2019 | May  2019 | June  2019 | July  2019 | August  2019 | September  2019 | October  2019 | November  2019 | December  2019 | January  2020 | February  2020 |
|  |  | 3 | 4 | 5 | 6 | 7 | 8 | 9 | 10 | 11 | 12 | 1 | 2 |
| Albumin | 49 | -1.32 | -0.24 | -0.56 | 0.49 | 0.34 | 1.18 | 1.7 | 4.13 | 0.59 | -1.16 | -0.28 | -2.99 |
| ALP | 234 | -4.65 | -3.66 | -4.21 | -3.41 | -3.28 | 12.22 | 6.69 | 6.25 | 6.24 | 5.92 | 4.66 | -12.39 |
| ALT | 114 | -1.84 | -0.8 | -0.84 | 0.28 | 0.44 | -0.11 | -2.22 | -2.22 | -2.16 | -3.26 | -2.27 | -2.38 |
| Amylase | 190 | -1.49 | -0.29 | -0.54 | 0.19 | -1.39 | -4.24 | -2.78 | -2.07 | -1.61 | -1.69 | -1.74 | 2.7 |
| Anti-Streptolysin O | 257 | -1.45 | -2.28 | -3.33 | -1.12 | 4.96 | 6.69 | 3.73 | 1.32 | 1.36 | 2.82 | 2.26 | 1.33 |
| AST | 136 | 0.03 | 2.01 | 1.52 | 2.11 | 2.57 | 1.58 | 0.62 | 1.37 | 1.39 | 0 | 1.44 | 9.09 |
| Bilirubin, direct | 2.51 | -1.41 | -0.48 | -1.12 | -0.53 | -1.16 | 0.35 | -2.12 | -1.8 | -1.84 | -2.2 | -1.2 | -0.07 |
| Bilirubin, Total | 3.83 | -0.11 | 0.07 | -0.72 | 1.31 | 1.77 | 3.37 | 1.46 | 2.2 | 3.18 | 2.46 | 2.47 | 2.84 |
| C Reactive Protein | 41.9 | -7.64 | -7.33 | -3.2 | 0.91 | -0.49 | 29.89 | -2.94 | -1.95 | -1.43 | -0.6 | -0.22 | -0.12 |
| Calcium | 13.6 | 1.7 | 1.33 | -0.53 | 0.63 | 0.1 | -3.04 | -2.97 | -1.33 | 0.25 | 1.03 | -0.05 | 1.38 |
| Chloride | 105.2 | 1.97 | 0.13 | -1.46 | -1.13 | -1.21 | -1.42 | -4.51 | -2.47 | -3.27 | -2.02 | -2.1 | -0.61 |
| Cholesterol, HDL | 73.8 | -0.09 | -0.01 | 4.6 | 4.79 | 3.26 | -12.72 | -3.37 | 3.2 | 3.94 | 0.64 | 1.44 | 11.31 |
| Cholesterol, LDL | 97.8 | -1.65 | 0.02 | 4.74 | 6.66 | -0.2 | -0.39 | -0.63 | -0.09 | 0.53 | -3.08 | -5.67 | -10.83 |
| Cholesterol, Total | 171 | -1.42 | 0.64 | 1.01 | 1.65 | 2.34 | -0.8 | 0.4 | -0.04 | 0.87 | 0.17 | -0.93 | -2.68 |
| Creatin Kinase | 268 | -0.8 | -0.46 | -0.99 | -1.17 | -0.1 | -0.43 | 0.32 | 1.03 | 1.04 | -0.31 | -0.56 | -2.14 |
| Creatinine | 4.09 | 0.26 | 1.31 | 2.08 | 2.47 | 0.09 | 2.36 | 1.92 | 3.41 | 3.43 | 2 | 1.37 | -0.67 |
| D-Dimer | 3.79 | -1.42 | -1.01 | -1.66 | -0.51 | 1.52 | 1.34 | 1.36 | -0.15 | 1.3 | 2.46 | 3.83 | 2.21 |
| GGT | 241 | 0.6 | 1.68 | 0.32 | 0.44 | 0.53 | 7.08 | -1.34 | -0.8 | 0.31 | -0.02 | -0.83 | -3.32 |
| Glucose | 232 | 0.23 | 0.94 | -0.04 | 0.27 | -0.21 | 1.44 | -0.6 | 0.18 | 0.89 | 0.43 | 0.54 | -0.4 |
| Hemoglobin A1c | 10.3 | -0.02 | 0.52 | 0.7 | -0.89 | 1.38 | 0.79 | 0.73 | 0.07 | 0.71 | 1.54 | 1.63 | 0.51 |
| Iron | 239 | 0.08 | 0.85 | 0.59 | 0.93 | 0.21 | 0.86 | 0.82 | -0.98 | 1.1 | 0.89 | 0.59 | -0.21 |
| LDH | 292 | -1.49 | 0.53 | 0.8 | 1.31 | 0.84 | 2.4 | 2.46 | 3.34 | 4.01 | 1.48 | 0.77 | 5.9 |
| Lipase | 98.7 | -1.61 | -0.84 | -1.3 | -1.85 | -2.59 | -1.07 | -2.43 | -2.46 | 0.04 | -1.45 | -2.51 | 14.78 |
| Lithium | 1.73 | 1.04 | 0.96 | 0.91 | 0.87 | -1.39 | 1.73 | -1.14 | -1.03 | -1.27 | 0.46 | 3.62 | 1.49 |
| Magnesium | 3.23 | -2.35 | -1.44 | -2.04 | -0.46 | -0.11 | 1.44 | 0.17 | -0.02 | 0.57 | -1.28 | 0.02 | 0.6 |
| Phosphorus | 7.38 | -1.49 | -1.51 | -2.34 | -0.33 | 0.73 | 4.34 | 1.85 | 2.28 | 2.85 | 1.41 | 2.08 | 3.39 |
| Potassium | 6.75 | -0.11 | 0.64 | 0.07 | 0.88 | 0.7 | 3.41 | 0.48 | 0.42 | 0.42 | -0.02 | 0.76 | -0.03 |
| Protein, Total | 75.6 | -2.38 | -1.79 | 0.31 | 1.69 | 0.05 | 1.7 | 0.28 | 1.11 | 2.12 | 1.51 | 2.26 | 0.86 |
| Rheumatoid factor | 51.3 | -0.61 | 1.92 | -2.17 | 1.59 | 0.21 | -2.16 | -2.09 | -0.68 | -0.23 |  | 1.07 | 3.21 |
| Sodium | 135 | 0.2 | 0.81 | 0.46 | 0.76 | 0.55 | 1.1 | 0.36 | 0.46 | 0.88 | 0.11 | 1.15 | 1.36 |
| Triglyceride | 226 | -2.05 | -1.16 | -1.74 | -0.09 | -0.62 | -6.1 | -2.04 | 0.25 | 0.63 | -0.83 | 0.24 | 6.35 |
| Urea | 117 | 0.92 | -0.31 | -2.19 | -1.71 | -3.03 | 1.57 | -1.15 | -0.8 | -1.15 | -2.79 | -2.89 | -3.78 |
| Uric Acid | 9.27 | -2.15 | -1.36 | 0.5 | 1.98 | 1.26 | 2.71 | -0.48 | -2.09 | -1.92 | -0.15 | -1.07 | -2.04 |
| 25-OH Vitamin D | 27.63 | -5.4 | -8.48 | -0.99 | 2.83 | -1.96 | -4.37 | -3.51 | -4.67 | -2.34 | 0.99 | -0.66 | 9.02 |
| AFP | 62.8 | -4.3 | -5.41 | -4.54 | -2.39 | -0.02 | -2.8 | -4.17 | -2.52 | -7.36 | -3.27 | -3.18 | -3.39 |
| CA 125 | 95.47 | 3.63 | 3.8 | -0.73 | -2.02 | -2.03 | -5.68 | -5.15 | -3.74 | -1.07 | -4.91 | -1.66 | -1.99 |
| CA 15-3 | 90.88 | -5.09 | -7.3 | -1.32 | -2.98 | 1.92 | -2.87 | -5.24 | -2.19 | -5.89 | 0.25 | 1.03 | 0.17 |
| CA 19-9 | 106.29 | -0.97 | 0.32 | 1.9 | 2.58 | 8.2 | 10.69 | 12.64 | -1.84 | 0.48 | 3.61 | 2.2 | 0.3 |
| CEA | 50.62 | -3.17 | -3.98 | -0.12 | -1.53 | 0.08 | -2.8 | -0.92 | -1.76 | -3.75 | -2.35 | -0.45 | -1.64 |
| Ferritin | 195.6 | -2.84 | -2.94 | -3.39 | 1.99 | 3.52 | -5.07 | -3.98 | 0.81 | -1.63 | 2.11 | -1.59 | -2.74 |
| Folate | 13.3 | 9.34 | 23.52 | 3.95 | 9.79 | 8.85 | 2.27 | -0.57 | 3.22 | 2.85 | -1.16 | 0.99 | 5.86 |
| Free T3 | 25.39 | -11.01 | 1.85 | 1.48 | 3.36 | 1.32 | 5.28 | -0.97 | 0.39 | 1.51 | -0.6 | -5.06 | 1.83 |
| Free T4 | 3.23 | -12.84 | -1.29 | -0.34 | -0.54 | -1.67 | 2.57 | -3.94 | -3.31 | -0.79 | 2.61 | -0.45 | -2.33 |
| FSH | 46.73 | 1.74 | 4.07 | 0.83 | 0.62 | 1.1 | 0.58 | -0.51 | -1.77 | -3.72 | -2.25 | 0.68 | -3.57 |
| hCG | 1106.5 | 0.42 | -0.08 | 0.02 | -1.53 | -2.5 | 1.85 | -2.26 | -4.92 | -5.27 | 18.2 | -2.05 | -2.93 |
| IgE | 287.58 | -9.48 | -1.47 | -0.83 | -1.03 | -0.01 | -1.52 | 1.81 | 4.54 | 7.08 | 6.53 | 3.44 | -1.85 |
| Insulin | 78.24 | -1.1 | -1.58 | -4.15 | -4.21 | -1.96 | 0.16 | 0.41 | -2.74 | -4.27 | -2.1 | -1.68 | 0.45 |
| LH | 48.3 | -1.03 | 0.81 | -0.82 | 0.47 | -1.28 | -4.34 | 3.61 | 1.42 | 1.31 | 0.46 | 0.72 | 2.97 |
| NT-ProBNP | 4550 | 4.55 | -1.17 | -3.29 | -0.53 | 4.6 | 0.87 | 2.1 | -4.12 | -1.69 | -4.16 | -3.97 | -1.26 |
| Estradiol | 556 | -11.25 | -7.39 | -7.09 | -8.13 | -6.88 | -3.69 | -8.36 | -6.86 | -7.98 | -11.25 | -14.69 | -7.76 |
| PTH | 183.55 | 8.67 | 6.95 | 6.54 | 4.39 | 7.82 | 8.15 | 8.7 | 5.24 | 4.88 | -2.66 | 2.94 | 1.97 |
| Procalcitonin | 9.15 | 0.39 | -3.87 | -3.56 | -1.23 | -3.7 | -3.91 | -5.54 | -6.11 | -3.54 | -0.94 | 0.29 | 5.21 |
| Prolactin | 820.44 | -2.09 | 0.01 | 0.19 | 0.71 | -0.96 | -1.66 | -2.31 | -2.4 | -2.86 | -0.5 | -0.4 | -0.6 |
| PSA, Free | 11.47 | -5.74 | -3.61 | 1.39 | 1.28 | 0.28 | -1.7 | 0.49 | -0.15 | -0.26 | 8.48 | 4.78 | 2.52 |
| PSA, Total | 36.88 | -2.88 | -2.35 | 5.34 | 2.01 | 0.48 | -1.4 | 0.12 | 0.53 | 0.79 | -1.13 | -1.98 | -3 |
| Testosterone | 2.51 | -21.88 | -5.73 | -3.63 | -6.77 | -6.32 | -5.04 | -4.62 | 2.85 | 3.67 | 1.27 | -3.92 | -5.04 |
| Troponin Ths | 2122 | 1.95 | 0.91 | 1.21 | 0.78 | 2.51 | -1.15 | -1.39 | -1.05 | 1.09 | 4.27 | 5.65 | 2.96 |
| TSH | 8.18 | -6.82 | 2.32 | 1.04 | 3.14 | 1.8 | 4.72 | 0.68 | -1.38 | -0.21 | -3.29 | 2.71 | 0.12 |
| Vitamin B12 | 958.64 | -1.94 | 4.36 | -0.34 | 0.79 | -0.09 | -1.02 | -4.56 | -0.73 | -4.6 | -3.55 | -0.2 | 4.03 |

Supplementary Table 4: Passing-Bablok regression analysis based on monthly the EQA results

| Analyte | Target Value  (IQC Level 1) | Target Value  (IQC Level 2) | Regression Equation |
| --- | --- | --- | --- |
| Albumin (g/L) | 32.7 | 49 | y = -0.18664 + 1.021x |
| ALP (U/L) | 94.9 | 234 | y = -2.06972 + 1.014x |
| ALT (U/L) | 48 | 114 | y = -0.32412 + 1.014x |
| Amylase (U/L) | 76.3 | 190 | y = -2.01413 + 1.013x |
| Anti-Streptolysin O (U/mL) | 122 | 257 | y = 5.29594 + 0.986x |
| AST (U/L) | 46.4 | 136 | y = -0.37633 + 1.035x |
| Bilirubin, Direct (µmol/L) | 17.00 | 42.92 | y = -0.06694 + 0.994x |
| Bilirubin, Total (µmol/L) | 17.96 | 65.49 | y = -0.37060 + 1.004x |
| C Reactive Protein (mg/L) | 7.96 | 41.9 | y = 0.67387 + 0.991x |
| Calcium (mmol/L) | 2.22 | 3.39 | y = -0.03946 + 1.027x |
| Chloride (mmol/L) | 76.5 | 105.2 | y = -4.10378 + 1.039x |
| Cholesterol, HDL (mmol/L) | 0.74 | 1.91 | y = -0.12435 + 1.051x |
| Cholesterol, LDL (mmol/L) | 1.45 | 2.53 | y = 0.01409 + 0.994x |
| Cholesterol, Total (mmol/L) | 2.35 | 4.42 | y = -0.00134 + 1.010x |
| Creatin Kinase (U/L) | 155 | 268 | y = -6.77097 + 1.018x |
| Creatinine (µmol/L) | 94.61 | 361.64 | y = -5.74375 + 1.050x |
| D-Dimer (µg/L) | 820 | 3790 | y = -22.35740 + 1.093x |
| GGT (U/L) | 53.3 | 241 | y = 0.28239 + 1.015x |
| Glucose (mmol/L) | 5.55 | 12.88 | y = -0.05839 + 1.011x |
| Hemoglobin A1c (%) | 5.7 | 10.3 | y = -0.21191 + 1.027x |
| Iron (µmol/L) | 18.80 | 42.78 | y = -0.85120 + 1.038x |
| LDH (U/L) | 170 | 292 | y = 0.50208 + 1.001x |
| Lipase (U/L) | 45.6 | 98.7 | y = 0.14841 + 0.97892x |
| Lithium (mmol/L) | 0.9 | 1.73 | y = 0.01097 + 1.005x |
| Magnesium (mmol/dL) | 0.81 | 1.33 | y = -0.01941 + 1.032x |
| Phosphorus (mmol/L) | 1.34 | 2.38 | y = -0.02559 + 1.019x |
| Potassium (mmol/L) | 3.66 | 6.75 | y = -0.07989 + 1.022x |
| Protein, Total (g/L) | 49.3 | 75.6 | y = -0.56485 + 1.029x |
| Rheumatoid factor (U/mL) | 20.5 | 51.3 | y = -0.56148 + 1.009x |
| Sodium (mmol/L) | 113 | 135 | y = -10.33204 + 1.079x |
| Triglyceride (mmol/L) | 1.37 | 2.55 | y = -0.00027 + 1.014x |
| Urea (mmol/L) | 14.03 | 41.78 | y = -0.31149 + 1.014x |
| Uric Acid (mmol/L) | 0.27 | 0.55 | y = -0.00690 + 1.012x |
| 25-OH Vitamin D (nmol/L) | 32.25 | 68.96 | y = 2.52773 + 1.068x |
| AFP (kIU/L) | 12 | 62.8 | y = -0.80798 + 1.091x |
| CA 125 (kIU/L) | 33.46 | 95.47 | y = -0.43970 + 1.092x |
| CA 15-3 (kIU/L) | 20.81 | 90.88 | y = -1.43687 + 1.08x |
| CA 19-9 (kIU/L) | 25.05 | 106.29 | y = -2.36323 + 1.108x |
| CEA (ug/L) | 5.17 | 50.62 | y = -0.19772 + 1.053x |
| Ferritin (ug/L) | 25.37 | 195.6 | y = -5.65467 + 1.121x |
| Folate (nmol/L) | 10.42 | 30.14 | y = -0.06045 + 1.060x |
| Free T3 (pmol/L) | 5.5 | 25.39 | y = -0.05642 + 1.04x |
| Free T4 (pmol/L) | 15.19 | 41.58 | y = 0.68498 + 0.994x |
| FSH (IU/L) | 18.51 | 46.73 | y = -0.72578 + 1.091x |
| hCG (IU/L) | 5.11 | 1106.5 | y = 0.10711 + 1.054x |
| IgE (kU/L) | 123.78 | 287.58 | y = -1.35938 + 1.075x |
| Insulin (mU/L) | 24.46 | 78.24 | y = -0.10647 + 1.051x |
| LH (IU/L) | 9.77 | 48.3 | y = -0.86649 + 1.06x |
| NT-ProBNP (ng/L) | 134 | 4550 | y = -1.54916 + 1.03x |
| Estradiol (pmol/L) | 385.46 | 2041.08 | y = 33.59584 + 0.997x |
| PTH (ng/L) | 51.6 | 183.55 | y = -5.26082 + 1.073x |
| Procalcitonin (ug/L) | 0.47 | 9.15 | y = -0.01403 + 1.042x |
| Prolactin (ug/L) | 10.49 | 38.56 | y = 0.05783 + 1.04x |
| PSA, Free (ug/L) | 0.85 | 11.47 | y = 0.01080 + 1.036x |
| PSA, Total (ug/L) | 3.93 | 36.88 | y = 0.17137 + 1.043x |
| Testosterone (nmol/L) | 20.13 | 8.71 | y = -0.03897 + 1.026x |
| Troponin Ths (ng/L) | 28.35 | 2122 | y = -0.99570 + 1.062x |
| TSH (mIU/L) | 1.38 | 8.18 | y = -0.02832 + 1.027x |
| Vitamin B12 (pmol/L) | 377.86 | 707.48 | y = -7.82296 + 1.058x |
